# Study of the Structural Chemistry of the Inclusion Complexation of 4-Phenylbutyrate and Related Compounds with Cyclodextrins in Solution: Differences in Inclusion Mode with Cavity Size Dependency

**DOI:** 10.3390/ijms242015091

**Published:** 2023-10-11

**Authors:** Kindness L. Commey, Akari Nakatake, Airi Enaka, Ryota Nakamura, Koji Nishi, Kenji Tsukigawa, Hirohito Ikeda, Koki Yamaguchi, Daisuke Iohara, Fumitoshi Hirayama, Keishi Yamasaki, Masaki Otagiri

**Affiliations:** 1 Faculty of Pharmaceutical Sciences, Sojo University, 4-22-1 Ikeda, Kumamoto 860-0082, Japan; g1971d03@m.sojo-u.ac.jp (K.L.C.); chii6natoriumu2sou@gmail.com (A.N.); g1851033@m.sojo-u.ac.jp (A.E.); g1951079@m.sojo-u.ac.jp (R.N.); knishi@ph.sojo-u.ac.jp (K.N.); tsukigawa@ph.sojo-u.ac.jp (K.T.); kyamag05@ph.sojo-u.ac.jp (K.Y.); dio@ph.sojo-u.ac.jp (D.I.); fhira@ph.sojo-u.ac.jp (F.H.); 2 DDS Research Institute, Sojo University, 4-22-1 Ikeda, Kumamoto 860-0082, Japan; 3 Faculty of Pharmaceutical Sciences, Fukuoka University, 8-19-1 Jonan-ku, Fukuoka 814-0180, Japan; ikeda@fukuoka-u.ac.jp

**Keywords:** cyclodextrins, 4-phenylbutyrate, inclusion complexation, structural chemistry, inclusion mode, cavity size dependency

## Abstract

4-phenylbutyrate (PB) and structurally related compounds hold promise for treating many diseases, including cancers. However, pharmaceutical limitations, such as an unpleasant taste or poor aqueous solubility, impede their evaluation and clinical use. This study explores cyclodextrin (CD) complexation as a strategy to address these limitations. The structural chemistry of the CD complexes of these compounds was analyzed using phase solubility, nuclear magnetic resonance (NMR) spectroscopic techniques, and molecular modeling to inform the choice of CD for such application. The study revealed that PB and its shorter-chain derivative form 1:1 αCD complexes, while the longer-chain derivatives form 1:2 (guest:host) complexes. αCD includes the alkyl chain of the shorter-chain compounds, depositing the phenyl ring around its secondary rim, whereas two αCD molecules sandwich the phenyl ring in a secondary-to-secondary rim orientation for the longer-chain derivatives. βCD includes each compound to form 1:1 complexes, with their alkyl chains bent to varying degrees within the CD cavity. γCD includes two molecules of each compound to form 2:1 complexes, with both parallel and antiparallel orientations plausible. The study found that αCD is more suitable for overcoming the pharmaceutical drawbacks of PB and its shorter-chain derivative, while βCD is better for the longer-chain derivatives.

## 1. Introduction

4-phenylbutyrate (PB) is a phenyl-substituted fatty acid derivative. It is used to manage urea cycle disorders (UCDs), a group of rare metabolic disorders caused by inborn deficiencies in the urea cycle and characterized by hyperammonemia [1,2,3,4]. PB exhibits many other biological activities, including acting as a low molecular weight chemical chaperone (LWCC) and histone deacetylase inhibitor, and its therapeutic effects against hemoglobinopathies, cancer, and cystic fibrosis are being investigated in clinical trials [5,6,7,8].

**Figure 1:**
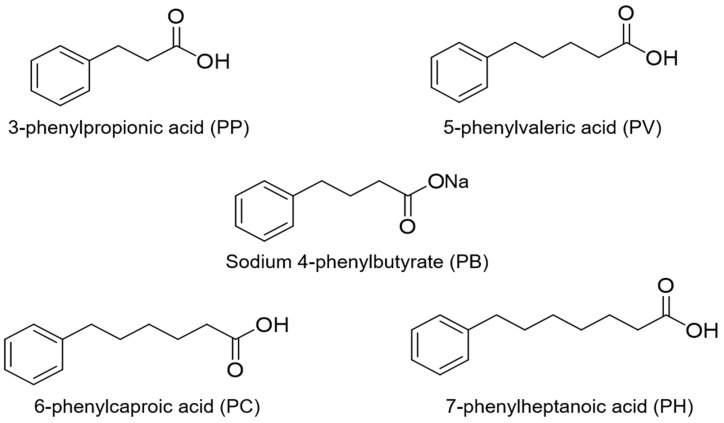
Chemical structures of PB and structurally related compounds (PP, PV, PC, and PH).

A series of structurally related compounds of PB (Figure 1) have been suggested to have similar biological activities as PB. In a study of PB and other LWCC in protecting human renal proximal tubule epithelial cells, the efficacy and potency of these compounds were shown to be attributable to the presence of a hydrophilic end followed by a long hydrocarbon, with the length of the hydrophobic hydrocarbon region correlating with potency [9]. A recent study of the structural chemistry of these compounds and their binding to human serum albumin at our laboratory showed that the binding affinities between the ligands and albumin were dependent on the number of methylene units between the phenyl and carboxylate groups on the molecule, and the maximum affinity was found for 6-phenylcaproic acid (number of methylene units 5) [10]. Therefore, PB and these structurally related compounds hold potential as lead compounds for the management of various diseases.

However, PB has a notoriously unpleasant taste, resulting in poor patient compliance. Furthermore, the related compounds have unfavorable pharmaceutical properties, such as poor aqueous solubility, oily physical state, or unpleasant odor and taste, impeding their evaluation and potential clinical use [5,11,12]. To fully explore the therapeutic potential of these promising compounds, it is necessary to investigate methods for addressing these limitations. One such approach is complexing these molecules with cyclodextrins (CDs). CDs are cyclic oligosaccharides comprising six, seven, eight, or more D-glucopyranose units linked by α-1,4-glycosidic bonds that form inclusion complexes with a wide range of molecules in a host:guest fashion [13,14,15]. This inclusion complex formation results in profound improvements in the physicochemical and biological properties of drug substances, including improving their solubility, bioavailability, and stability, as well as masking unpleasant odors and tastes and converting liquids and oils to free-flowing powders [16,17,18]. Moreover, due to their favorable toxicological profile, CDs are preferred over organic solvents for in vitro/in vivo evaluation of new chemical entities [16]. Therefore, we explored CD complexation as a strategy to overcome the pharmaceutical drawbacks of PB and its structurally related compounds. From our studies, we recently reported that αCD significantly masks the unpleasant taste of PB and can address the limitations of current market formulations [19].

Thus, in our continuing investigations, we undertook structural chemistry analyses of the PB-related compounds and their complex formation with CDs in solution using phase solubility studies, nuclear magnetic resonance (NMR) spectroscopic techniques, and molecular modeling. Though some studies on the complexation of some phenyl alkanoates with CDs have been reported, the present study provides insight into the CD cavity size dependency and the effect of guest structure on the stability, inclusion mode, and stoichiometry of CD inclusion complexes of PB and its therapeutically relevant and structurally related compounds in aqueous solution [20,21,22].

The findings of the present study provide the basis for selecting the most appropriate CDs for complexing PB-related compounds to overcome their pharmaceutical limitations and allow for their clinical evaluation and use.

## 2. Results and Discussion

### 2.1. Phase Solubility Studies

**Figure 2:**
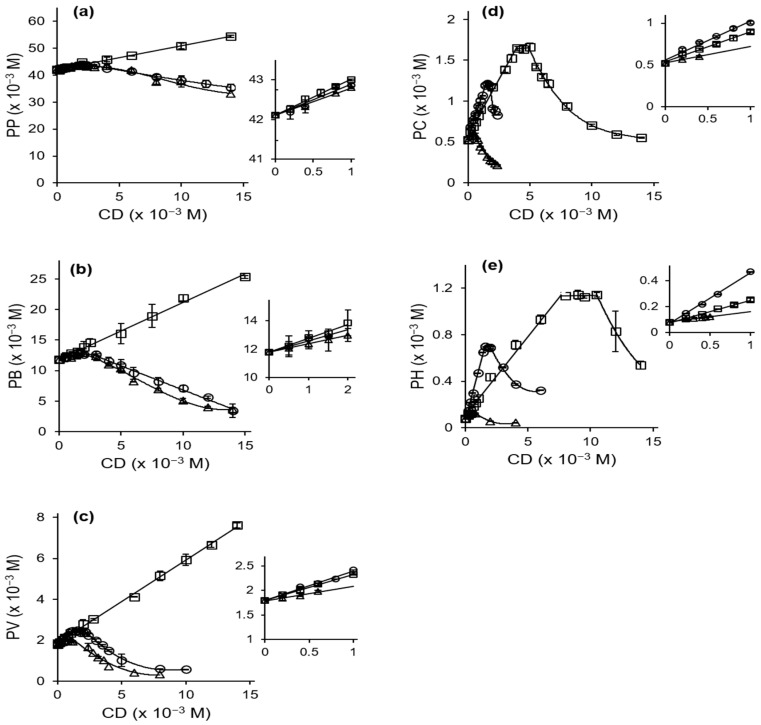
Phase solubility diagrams of (**a**) PP–CD; (**b**) PB–CD; (**c**) PV–CD; (**d**) PC–CD; (**e**) PH–CD systems in 0.1 M phosphate buffer (pH 2.1) at 25 °C. Each point represents the mean ± SD (n = 3). Open square; αCD, open circle; βCD, open triangle; γCD. Data used to construct (**b**) are from our previous work [19].

**Figure 3:**
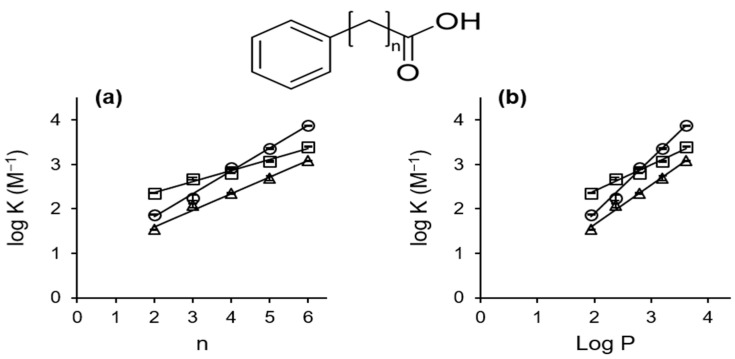
Relationship between (**a**) log K and number (n) of -CH_2_- groups; (**b**) log K and partition coefficient (Log P) of PB and structurally related compounds. Each point represents the mean ± SD (n = 3). Log P values were estimated by Chem Bio Draw. Open square; αCD, open circle; βCD, open triangle; γCD).

Figure 2 shows the phase solubility diagrams of the interactions between the CDs and PB or related compounds. αCD showed A_L_-type phase solubility diagrams with PP, PB, and PV; however, it showed B_S_-type diagrams for PC and PH. βCD and γCD showed B_S_-type diagrams with PB and all of the related compounds. Table 1 shows the guest/host ratios of the systems derived from analyses of their phase solubility diagrams. The results indicate that PP, PB, and PV form 1:1 complexes, whereas PC and PH form 1:2 (guest:host) complexes with αCD. Moreover, all of the guest compounds form 1:1 and 2:1 (host:guest) complexes with β and γCD, respectively [23]. The apparent stability constants (K), estimated from the initial linear portion of the diagrams, are shown in Table 2. The complex stability trend was βCD > αCD > γCD for all the guest compounds except for PP and PB, where the αCD complexes were the most stable. The stability constants for the PP and PB systems agree reasonably with the values from previous reports [20,24]. The apparent stability constants increased as the guest methylene chain length increased. Moreover, there was a strong positive correlation between log K and the partition coefficient, Log P, of the compounds (correlation coefficient, R^2^ > 0.982 for all the CD systems) (Figure 3a,b). These findings suggest that hydrophobic interactions play a critical role in the stability of the complexes [21].

### 2.2. ^1^H NMR Spectroscopy

#### 2.2.1. ^1^H NMR Chemical Shift Changes

Table 3 shows the changes in the chemical shift of PB or related compounds in the presence of equimolar amounts of CDs. The αCD systems mostly showed downfield changes, while the β and γCD systems had the opposite effect. This indicates that the guest compounds may have different orientations within the different CD complexes [25]. Furthermore, in the β and γCD systems, the aromatic protons showed larger displacements compared to the alkyl protons, while the opposite was observed in the αCD systems. This suggests that the size of the CD cavity affects the orientation and disposition of the guest compounds. A certain specificity of CDs with respect to inclusion complex formation has been recognized in the early work of CD research, where the guest molecule must fit at least partially into the CD cavity [15,26]. The results indicate that the smaller αCD cavity preferentially includes the alkyl chain of the PB and related compounds and only partially includes the aromatic ring, while both the alkyl chain and the aromatic ring are deeply included in the larger βCD and γCD cavities. This assertion is supported by the changes in the chemical shift of the CDs in the presence of equimolar amounts of the guest compounds, as shown in Table 4. Typically, the inner H3′ and H5′ protons of CDs in inclusion complexes experience shift changes due to the hydrophobic or ring current effect of guest compounds [26]. The H3′ proton of αCD showed significant upfield shift changes with all the guest compounds, while the H5′ proton showed negligible changes with PP, PB, and PV but significant changes with PC. This indicates that PC and PH, which have longer alkyl chains, are more deeply included in the αCD cavity compared to PP, PB, and PV [27]. On the other hand, the guest compounds caused upfield shift changes in the H3′ and H5′ protons of both the βCD and γCD systems. However, the changes were more significant in the βCD system. This indicates that the guest compounds fit better in the βCD cavity despite being included deeply in both CD cavities. However, considering the case with PP and PB, whose αCD complexes were more stable, it is important to note that even though the βCD cavity appears to be the most suitable in terms of spatial fit, this does not necessarily imply that the βCD complex will be the most stable for all the guest compounds. Other factors, such as the optimization of the host–guest interaction distance and entropic changes, may result in a less optimal fit being more stable [28].

#### 2.2.2. Stoichiometry and Inclusion Equilibrium

**Figure 4:**
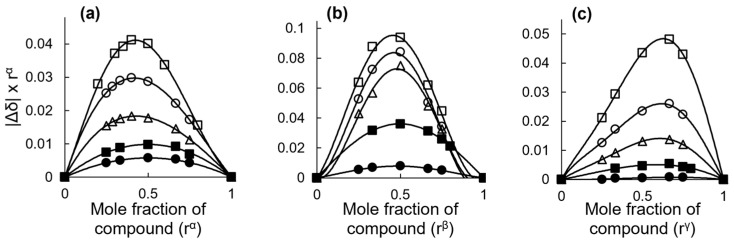
Continuous variation plots of the ^1^H NMR chemical shift changes for the inclusion complexation of PB and related compounds with (**a**) αCD; (**b**) βCD; and (**c**) γCD in 0.1 M sodium borate/D_2_O at 25 °C. The chemical shift of proton Y was monitored for the αCD systems, whereas proton X was monitored for the β and γCD systems. The total concentration of PB (or related compound) and CDs was 1.0 × 10^−2^ M (2.0 × 10^−2^ M for the PH-αCD system). Closed circle: PP, closed square: PB, open triangle: PV, open circle: PC, open square: PH. Data used to construct the PB plots are from our previous work [19].

Figure 4 shows the continuous variation plots for the inclusion complexation of PB and related compounds with the CDs, obtained by monitoring the chemical shift changes of proton Y for the αCD systems and proton X for the βCD and γCD systems. The total concentration of the guests and CDs was kept constant at 1.0 × 10^−2^ M (1.0 × 10^−2^ M for the PH-αCD system). For the αCD systems (Figure 4a), PP and PB achieved maxima at 0.5 guest/(guest + host) mole fraction, whereas PV, PC, and PH achieved maxima at 0.35. This suggests a tendency of the stoichiometry to change from 1:1 toward 1:2 (guest:host) as the number of methylene units increases beyond 3 (PB). On the other hand, the βCD and γCD systems (Figure 4b,c) achieved maxima at a 0.5 and 0.67 guest/(guest + host) mole fraction, respectively, for all of the guest compounds. These results confirm that βCD and γCD with relatively larger cavity sizes form 1:1 and 2:1 (guest:host) inclusion complexes, respectively, with the guest compounds. The stoichiometry of CD inclusion complexes usually obeys the law of constant proportions, i.e., the binding molar ratio of guest/host are integral with each other. This implies that when a guest molecule is too large to be included in one CD cavity, or the host cavity is too small to form inclusion complexes with a guest molecule, more than one CD is available for the inclusion complexation [15,26]. For instance, Utsuki et al. previously reported that tranilast, a cinnamic acid derivative, forms inclusion complexes of 1:2, 1:1, and 2:1 stoichiometries with α, β, and γCD, respectively, in aqueous solution [29]. Therefore, the obtained complex stoichiometries of guest compounds with the CDs are consistent with this inclusion complexation behavior.

**Figure 5:**
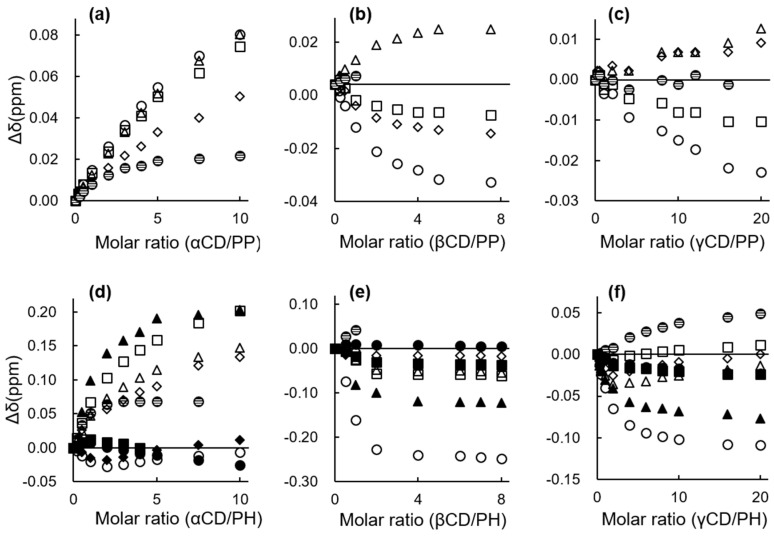
^1^H NMR chemical shift changes of PP and PH as a function of the concentration of (**a**,**d**) αCD; (**b**,**e**) βCD; and (**c**,**f**) γCD in 0.1 M sodium borate/D_2_O at 25 °C. The changes in chemical shifts are expressed as Δδ = δ _with CD_ − δ _without CD_. The concentration of PP was 5.0 × 10^−3^ M for all CD systems, whereas the concentration of PH was 5.0 × 10^−3^ M for the αCD system, and 2.5 × 10^−3^ M for the β and γCD systems. Proton key: Open triangle: A, open diamond: B, closed triangle: C, closed diamond: D, closed square: E, closed circle: F, open circle: X, open square: Y, checked circle: Z.

Figure 5 shows the chemical shift displacement of PP and PH protons as a function of CD concentration. For the PP-αCD system (Figure 5a), all PP protons shifted downfield regardless of CD concentration. However, for the PH-αCD system (Figure 5d), protons D and X shifted upfield, while E and F shifted downfield at low CD concentrations. Notably, these directions were reversed at higher CD concentrations. These biphasic shift changes could be attributed to the CD concentration-dependent change in stoichiometry of the PH-αCD complex [27]. This supports the earlier assertion that the stoichiometry shifts from 1:1 to 1:2 (guest:host) as the alkyl chain length increases. For the βCD systems, proton A was displaced downfield for PP (Figure 5b) but upfield for PH (Figure 5e). This suggests a slight difference in the orientation of the guest compounds within the βCD cavity as the alkyl chain length increases from PP to PH [25]. However, no biphasic shift changes were observed in either the PP or PH systems, confirming that the guest compounds, independent of alkyl chain length, form 1:1 complexes with βCD. In the γCD systems, PP protons X and Y shifted downfield at low CD concentrations, but were reversed at higher concentrations (Figure 5c). PH protons A, B, and Y showed similar behavior (Figure 5f). These suggest a CD concentration-dependent change in the stoichiometry of the γCD complexes and confirm that the guest compounds form 2:1 (guest:host) complexes with γCD, regardless of the alkyl chain length [27]. 

The stability constants (K_1:1_) of the PH–CD complexes estimated by analyzing the first-order dependences of the chemical shift change of PH protons on CD concentration were 123 ± 2 M^−1^ (determined from protons A and Y), 2513 ± 469 M^−1^ (A and X), and 370 ± 6 M^−1^ (C and X) for the αCD, βCD, and γCD complexes, respectively. These values represent a 20-fold reduction for the αCD complex and a 3-fold reduction for the βCD and γCD complexes, compared to the estimated values at pH 2.1 (obtained from phase solubility studies). This indicates that the ionization of PH (free acid) has a greater destabilizing effect on the αCD complex than on the βCD and γCD complexes. A possible explanation is that the ionized acid is highly hydrated, making it less compatible with the smaller cavity of αCD [21].

#### 2.2.3. 2D ROESY Spectroscopy

**Figure 6:**
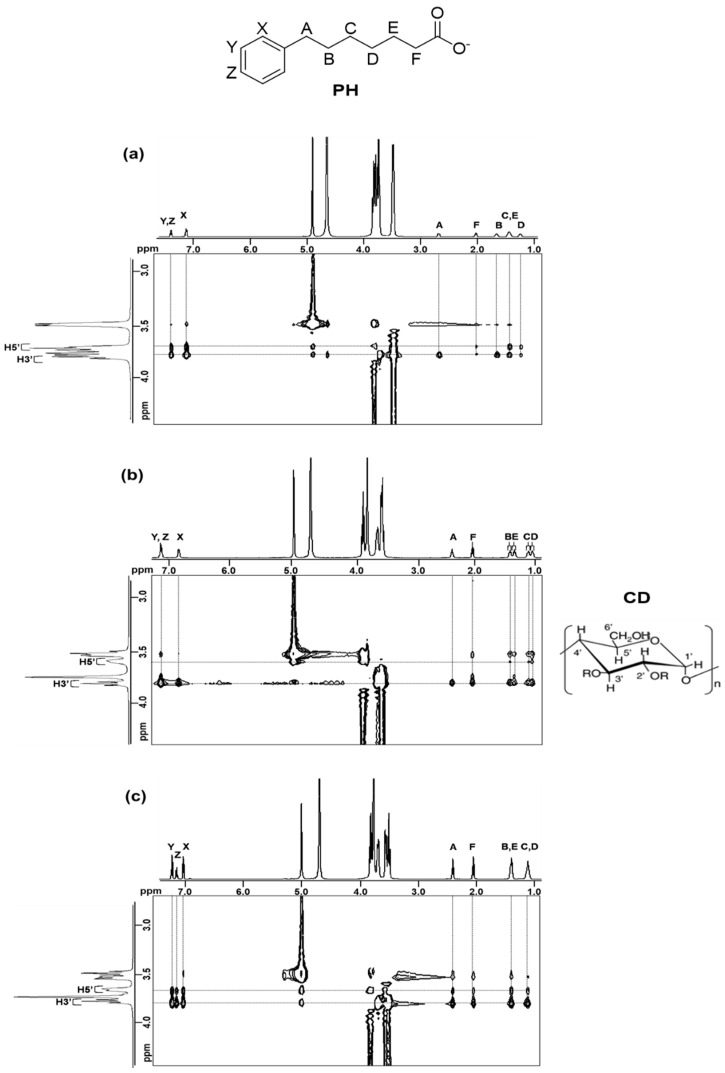
Partial contour plots of the ROESY spectra of (**a**) PH-αCD; (**b**) PH-βCD; and (**c**) PH-γCD in 0.1 M sodium borate/D_2_O at 25 °C. The concentration of the guest and the host were 2.5 × 10^−2^ M/1.0 × 10^−1^ M (PH/αCD), 1.5 × 10^−2^ M/2.5 × 10^−2^ M (PH/βCD), and 2.5 × 10^−2^ M/2.5 × 10^−2^ M (PH/γCD).

Two-dimensional ROESY studies were conducted on the PH–CD systems to elucidate the inclusion structures of the complexes. Figure 6 shows the partial contour plots of the 2D ROESY spectra of the PH–CD systems. For the αCD system (Figure 6a), correlation peaks were observed between the CD inner H3′ and H5′ protons and all the PH protons, except between protons A and B and the H5′ proton. This implies that the alkyl chain of PH is deeply included in the αCD cavity, entering from the secondary hydroxyl end and traversing the CD cavity, with the carboxylate moiety deposited just outside the primary hydroxyl end of the cavity. This appears reasonable since molecular models indicate that about six methylene groups threaded through a CD cavity essentially fill the cavity [30]. The aromatic ring of PH is deposited around the secondary hydroxyl end of the CD cavity and is shallowly included by a second αCD molecule to form a 1:2 PH-αCD inclusion complex. For the βCD system (Figure 6b), correlation peaks between the inner H3′ proton and all the PH protons were observed. Additionally, the inner H5′ protons showed correlation peaks with PH protons B, C, and D. This indicates that the aromatic ring of PH is deeply included in the βCD cavity with the alkyl chain bending at the mid-section (B, C, and D) within the CD cavity. The terminal section (E, F) of the alkyl chain and the carboxylate moiety point back into the CD cavity around the secondary hydroxyl end of the cavity. In the case of the γCD system (Figure 6c), correlation peaks between the inner H3′ and H5′ protons of the CD and all of the PH protons were observed. This result suggests that two PH molecules are deeply included in the large γCD cavity, possibly entering from either end of the CD cavity and aligned in an antiparallel or parallel orientation to each other. Considering that host–guest inclusion complex formation is a dynamic process, these two modes of inclusion may exist simultaneously [31].

### 2.3. Molecular Modeling

**Figure 7:**
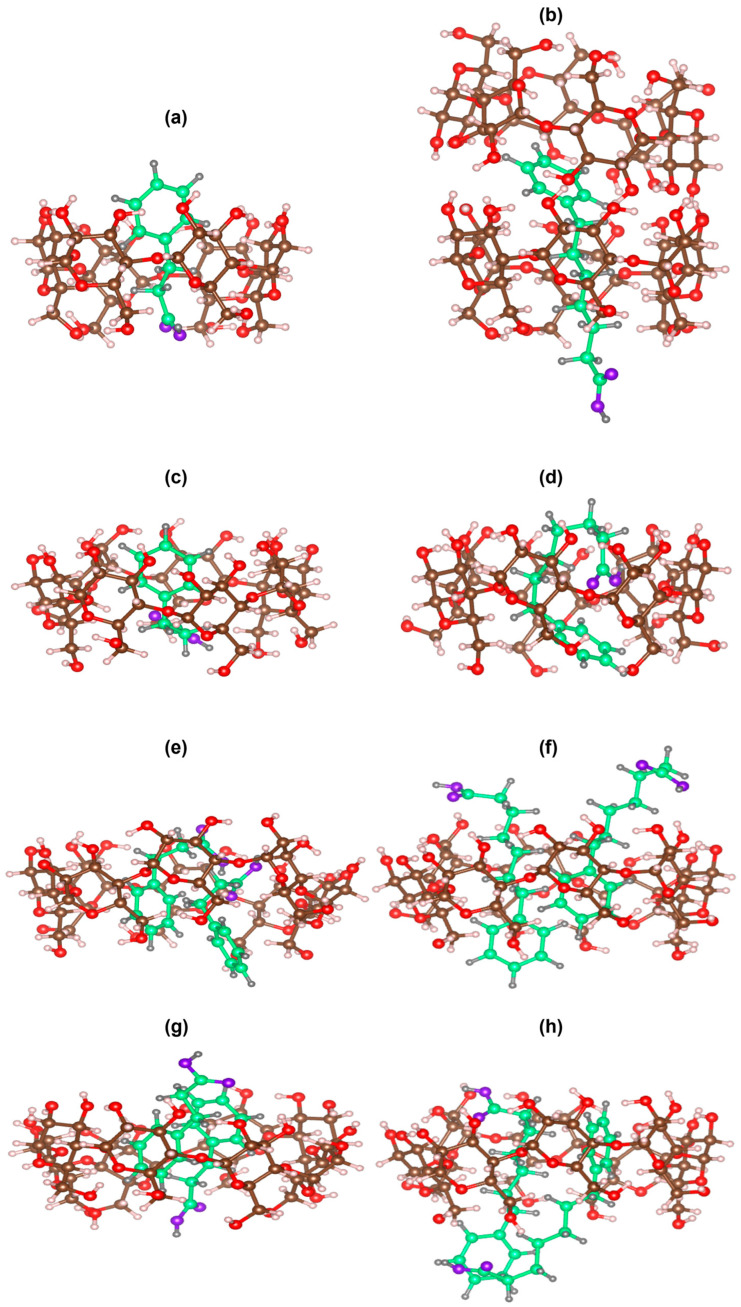
Possible inclusion structures of PP–CD and PH–CD complexes estimated by molecular docking model calculation: (**a**) PP–αCD; (**b**) PH–αCD; (**c**) PP–βCD; (**d**) PH–βCD; (**e**) PP–γCD parallel orientation; (**f**) PH–γCD parallel orientation; (**g**) PP–γCD antiparallel orientation; and (**h**) PH–γCD antiparallel orientation. The relative molecular sizes of the complexes are arbitrary. The green ball and stick represent the guest compound (PP or PH). The upper and lower sides of the CDs are the secondary and primary rims, respectively.

To obtain reasonable structural representations of the inclusion complexes of PP and PH with the CDs, molecular modeling was performed. The resulting structures are presented in Figure 7. In the PP-αCD complex (Figure 7a), the alkyl chain and carboxylate moiety are located inside the CD cavity, while the phenyl ring is at the rim of the secondary hydroxyl end. For the PH–αCD complex (Figure 7b), the alkyl chain is inside the CD cavity, with the primary methylene and carboxylate moieties protruding from the primary end of the cavity, while the phenyl ring is located just outside the rim of the secondary end and is shallowly included by another αCD molecule that approaches with its secondary end. On the other hand, for the βCD complexes, PP is included inside the CD cavity with the phenyl ring located towards the secondary end, while the alkyl chain slightly bends within the CD cavity, as shown in Figure 7c. For the PH–βCD complex (Figure 7d), both the phenyl ring and alkyl chain of PH are located within the CD cavity, with the phenyl ring oriented towards the primary end of the cavity and the alkyl chain bending significantly and leaving the carboxylate moiety around the secondary rim of the cavity. For the γCD complexes, two inclusion modes each were calculated for PP (Figure 7e,g) and PH (Figure 7f,h), where two molecules of PP or PH are included in the large γCD cavity in a parallel or antiparallel orientation. The MOE-calculated structures agree reasonably with the results of the NMR spectroscopic studies despite using the unionized forms of the guest compounds for the calculation. The inclusion complex structures of the ionized forms in water may be almost identical to those of the unionized forms using MOE, as shown in the predicted structure of ionized PH with βCD in water using the density functional theory (DFT) (Figure S1) [32,33]. This predicted structure appears almost the same as the structure of the unionized form (Figure 7d). The only difference between the two structures is the degree of bending. The unionized form bends more into the CD cavity to increase complex stability.

## 3. Materials and Methods

### 3.1. Materials

PB, 5-phenylvaleric acid (PV), and 6-phenylcaproic acid (PC) were obtained from Tokyo Chemical Industry Co., Ltd. (Tokyo, Japan). 3-phenylpropionic acid (PP) was purchased from Wako Pure Chemical Industries Ltd. (Osaka, Japan). 7-phenylheptanoic acid (PH) was sourced from Alfa Aesar (Heysham, UK). αCD, βCD, and γCD were purchased from Nacalai Tesque Inc. (Kyoto, Japan). All other chemicals were obtained from commercial sources and were of the highest analytical grade.

### 3.2. Methods

#### 3.2.1. Phase Solubility Studies

Phase solubility studies were conducted according to the method described by Higuchi and Connors [23]. Briefly, 1 mL of CD solutions (0 to 14 mM, pH 2.1) were added to excess amounts of PB or related compounds placed in screw cap tubes. The resulting samples were shaken for 72 h at 25 °C and 120 rpm (Multi Shaker MMS-3020 in a temperature control chamber FMC-1000; Eyela Co., Ltd., Tokyo, Japan). The resulting suspensions were filtered through 0.2 µm membrane filters (Minisart RC 4, Sartorius Stedim Lab Ltd., Stonehouse, UK) and diluted appropriately. The solubility of PB or related compounds was determined by HPLC, and the data were used to construct phase solubility diagrams. The guest/host ratios for the systems showing B_S_-type solubility diagrams were estimated as the quotient of the amount of undissolved guest at the start of the plateau region and the CD concentration range corresponding to the plateau region [23]. The stability constants (K_1:1_) of the interactions, assuming the formation of 1:1 complexes, were also calculated according to Equation (1) [23]:

(1) $$K_{1:1}=\frac{\mathrm{Slope}}{S_{0}\left(1-\mathrm{Slope}\right)}$$

where S_0_ is the intrinsic solubility (solubility in the absence of CD) of PB or related compound at 25 °C, and the slope is the slope of the initial linear portion of the respective phase solubility diagrams.

##### HPLC Conditions

HPLC measurements were performed according to a previous report using a JASCO HPLC system (Jasco Corp., Tokyo, Japan) [10]. A YMC-PACK ODS AM 303 column (5 µm, 250 mm × 4.6 mm, YMC Co., Kyoto, Japan) was used as the stationary phase and was maintained at 40 °C. A linear gradient elution system was employed with a mobile phase comprised two solvents, A (0.05 M sodium dihydrogen phosphate) and B (0.05 M sodium dihydrogen phosphate, and acetonitrile (30:70, *v*/*v*)), programmed for PP, PB, and PV as follows: 0–7 min (30–100% B), 7–10 min (100% B), 10–15 min (100–30% B). For PC and PH, the elution program used was 0–7 min (50–100% B), 7–10 min (100% B), and 10–15 min (100–50% B) at a constant flow rate of 1 mL/min. A detection wavelength of 210 nm was used and monitored for 15 min for each sample, with retention times of 7.4, 8.9, 9.6, 9.4, and 10.7 min for PP, PB, PV, PC, and PH, respectively.

#### 3.2.2. ^1^H NMR Spectroscopy

^1^H NMR spectra were obtained using a JEOL-A500 spectrometer (Tokyo, Japan) operating at 500 MHz in 5 mm sample tubes at 25 °C using D_2_O/0.1 M sodium borate (pH meter reading of 9.4) as solvent. The resonance at 4.68 to 4.75 ppm, due to residual solvents (H_2_O and HOD), was used as an internal reference. Chemical shifts are given as parts per million (ppm), with an accuracy of ±0.001. No external reference was used to avoid possible interactions with the CDs. The ^1^H NMR signals of the guests (PB and related compounds) were assigned by 2D ^1^H-^1^H correlation spectroscopy (COSY), whereas those of the CDs were assigned according to a previous report [34]. The complex stoichiometries were determined by the continuous variation method, where the total concentrations of CD and guest were kept constant at 1.0 × 10^−2^ M. In addition, the changes in the chemical shift of CDs and the guest protons in their equimolar systems (5.0 × 10^−3^ M) were monitored. Due to the low solubility of the longer-chain guest compounds and their CD complexes, a medium of pH 9.4 was used to obtain sufficiently high concentrations of the complexes needed for ^1^H NMR measurements, particularly for the 2D ROESY NMR, which has a low sensitivity [35]. The CD concentration dependence of the chemical shift changes and stoichiometry of the complexes were studied by performing molar ratio titrations using PP and PH, where the concentration of PP or PH was maintained constant (5.0 × 10^−3^ M) while changing the concentration of the CDs [36]. The stability constant values of the complexes formed by PH were determined from the curves. Two-dimensional ROESY NMR experiments were performed for the CD-PH systems in the phase-sensitive mode using the same spectrometer. Each spectrum consisted of a matrix of F2 by F1 covering a sweep width of 5000 Hz with 36 scans. The spin-lock mixing time was 800 ms, with a relaxation delay of 4 s, and a 90° pulse width of 11.8 µs. The concentration of guest and host were 2.5 × 10^−2^ M/1.0 × 10^−1^ M (PH/αCD), 1.5 × 10^−2^ M/2.5 × 10^−2^ M (PH/βCD), and 2.5 × 10^−2^ M/2.5 × 10^−2^ M (PH/γCD). The stability constants (K_1:1_) of the PH–CD complexes were estimated by analyzing the first-order dependences of the chemical shift change of PH protons on CD concentration using Equation (2) [37]:

(2) $$\delta_{\mathrm{obs}}=\frac{\delta_{0}+\delta_{1}K_{1:1}{\left[\mathrm{CD}\right]}_{f}}{1+K_{1:1}{\left[\mathrm{CD}\right]}_{f}}$$

where δ_0_ and δ_obs_ are chemical shifts of PH protons without or with CD, respectively, and δ1 is the chemical shift of PH protons in the 1:1 complex. 

The equation was analyzed by the iteration method since the concentration of free CD ([CD]_f_) is unknown unless K_1:1_ had been determined beforehand. Moreover, the total CD concentration ([CD]_t_) was not high enough to ignore the concentration of the CDs in complex under the experimental conditions. Therefore, by setting [CD]_f_ = [CD]_t_ as a first approximation, Equation (2) was analyzed by a nonlinear least-squares method, and, in turn, [CD]_f_ values were calculated using the obtained apparent K_1:1_ value. This procedure was repeated until the K_1:1_ value converged at a constant value.

#### 3.2.3. Molecular Modeling

Molecular docking of PP and PH to CDs was performed using the molecular operating environment, MOE version 2019 (Chemical Computing Group Inc., Montreal, QC, Canada) [19]. According to the company’s recommendations, the Amber10: EHT force field was used for energy minimization. Crystal structures of CDs were obtained from the Protein Data Bank (PDB entry codes: 5E6Y, 2V8L, and 2ZYK for α, β, and γCD, respectively) and were used in the molecular docking studies. All ligand molecules in the PDB structures were eliminated for the docking study. Hydrogen atoms were added with the appropriate geometry, and their energies were minimized. The docking of PP and PH as ligands into CDs as receptors was conducted with the default values for the parameters. The docking scores were ranked by the parameter S score which is calculated using the London dG scoring function. A pose with the highest docking score (i.e., the lowest S) was chosen as the optimum docking pose. For the docking of PH to αCD, a molecule of PH was sandwiched by two αCD molecules, considering the most probable inclusion modes estimated by the NMR study. Additionally, for the docking of PP and PH to γCD, two molecules of each compound were inserted consecutively since the hydrophobic cavity of γCD is large enough to accommodate two molecules, as indicated by the NMR study. DFT calculations were also performed to verify the inclusion complex structures. This is described in the Supplementary Information.

## 4. Conclusions

In this study, the structural chemistry of the CD complexes of PB and its therapeutically relevant, structurally related compounds were analyzed to inform on the choice of CD for addressing the pharmaceutical limitations of these compounds. The study’s findings reveal the CD cavity size dependency of the stability, inclusion mode, and stoichiometry of the complexes. The smaller αCD forms more stable and soluble 1:1 complexes with bitter-tasting PB and its foul-smelling shorter-chain derivative (PP). Thus, αCD would be useful for masking their unpleasant organoleptic properties. In contrast, the βCD cavity size is ideal for the longer-chain PB-related compounds such as PC and PH, which are poorly soluble viscous oils. βCD forms stable 1:1 complexes with these compounds, implying a less bulky formulation compared to αCD, which forms less stable 1:2 (guest:host) complexes. Thus, βCD would be useful for obtaining free-flowing complex powders of these longer-chain compounds, albeit with limited solubility. γCD forms less stable 2:1 complexes of limited solubility with all of the compounds and, therefore, would be undesirable for overcoming the limitations of PB and related compounds. These findings using the natural CDs provide the basis for expanding the study to include CD derivatives such as 2-hydroxypropyl-βCD and sulfobutylether-βCD, which are known to form more soluble complexes and may prove more effective for overcoming the limitations of PB and related compounds.

## Figures and Tables

**Table 1:** Guest/Host ratios of PB and structurally related compounds with the natural CDs derived from analyses of the diagrams from phase solubility studies in 0.1 M phosphate buffer (pH 2.1) at 25 °C.

Compound	Guest/Host Ratio		
	αCD	βCD	γCD
PP	― ^a^	1.03 ± 0.08	1.97 ± 0.18
PB	― ^a^	1.02 ± 0.05 ^b^	2.14 ± 0.08 ^b^
PV	― ^a^	0.99 ± 0.05	2.21 ± 0.03
PC	0.55 ± 0.03	1.08 ± 0.01	2.30 ± 0.03
PH	0.44 ± 0.01	1.02 ± 0.01	2.20 ± 0.01

^a^ A_L_-type diagram (guest/host ratio = 1). ^b^ Data from our previous work [19]. The values are mean ± SD (n = 3).

**Table 2:** Apparent stability constant (K) of PB and structurally related compounds with the natural CDs in 0.1 M phosphate buffer (pH 2.1) at 25 °C.

Compound	K (M^−1^)		
	αCD	βCD	γCD
PP	226 ± 5	74 ± 3	34 ± 1
PB	481 ± 26 ^a^	178 ± 23 ^a^	119 ± 9 ^a^
PV	639 ± 20	838 ± 52	223 ± 7
PC	1185 ± 35	2283 ± 134	499 ± 59
PH	2500 ± 50	7458 ± 52	1213 ± 18

^a^ Data from our previous work [19]. The values are mean ± SD (n = 3).

**Table 3:** Changes in ^1^H NMR chemical shifts of PB and related compounds (5.0 × 10^−3^ M) in the presence of the natural CDs (5.0 × 10^−3^ M) in 0.1 M sodium borate/D_2_O at 25 °C.

		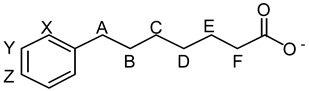 Change in Chemical Shift, Δδ (with CD − without CD) (ppm)								
CD	Compound	A	B	C	D	E	F	X	Y	Z
αCD	PP	0.010	0.006					0.012	0.012	0.006
	PB	0.028	0.026	0.002				0.020	0.020	― ^a^
	PV	0.044	0.045	― ^a^	−0.025			0.008	0.033	0.022
	PC	0.050	0.046	0.066	−0.013	−0.006		0.000	0.046	― ^a^
	PH	0.041	0.044	0.096	−0.015	0.009	0.004	−0.022	0.061	0.040
βCD	PP	0.009	−0.008					−0.016	−0.005	0.003
	PB	0.004	−0.003	−0.029				−0.072	−0.028	― ^a^
	PV	−0.016	0.009	−0.037	−0.014			−0.150	−0.056	0.026
	PC	−0.032	−0.007	−0.061	−0.019	−0.015		−0.168	−0.050	0.020
	PH	−0.054	−0.023	−0.086	−0.028	−0.029	−0.001	−0.188	−0.049	0.034
γCD	PP	0.003	0.002					−0.001	0.001	0.002
	PB	−0.001	0.003	−0.003				−0.009	−0.005	−0.002
	PV	−0.011	−0.004	−0.006	0.002			−0.026	−0.006	0.000
	PC	−0.031	−0.018	−0.023	−0.005	0.000		−0.047	−0.008	−0.001
	PH	−0.067	−0.055	−0.048	−0.042	−0.021	−0.014	−0.087	−0.020	−0.003

^a^ Could not be determined due to overlap with other signals.

**Table 4:** Changes in ^1^H NMR chemical shifts of the natural CDs (5.0 × 10^−3^ M) in the presence of PB and related compounds (5.0 × 10^−3^ M) in 0.1 M sodium borate/D_2_O at 25 °C.

		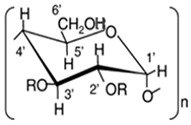 Change in Chemical Shift, Δδ (with Compound − without Compound) (ppm)				
CD	Proton	PP	PB	PV	PC	PH
αCD	H1′	−0.005	−0.003	−0.001	0.005	0.009
	H2′	−0.006	−0.005	−0.007	−0.011	−0.007
	H3′	−0.016	−0.025	−0.032	−0.053	−0.060
	H4′	−0.004	−0.006	0.003	― ^a^	― ^a^
	H5′	0.001	0.008	0.003	−0.014	― ^a^
	H6′a	0.005	−0.004	−0.005	−0.007	−0.008
βCD	H1′	−0.009	−0.015	−0.017	−0.016	−0.014
	H2′	0.001	−0.006	−0.009	−0.011	−0.008
	H3′	−0.022	−0.042	−0.053	−0.061	−0.061
	H4′	0.005	0.003	0.009	0.022	0.033
	H5′	−0.063	−0.122	−0.172	−0.206	−0.211
	H6′a	0.009	−0.004	−0.021	−0.032	−0.040
γCD	H1′	−0.002	−0.007	−0.008	−0.006	−0.006
	H2′	−0.002	−0.003	−0.005	−0.003	−0.006
	H3′	−0.004	−0.006	−0.008	−0.016	−0.028
	H4′	−0.002	−0.002	−0.002	−0.001	−0.001
	H5′	― ^a^	0.000	−0.009	−0.032	−0.055
	H6′a	−0.003	−0.007	−0.014	−0.015	−0.022

^a^ Could not be determined due to overlap with other signals. Chemical shift changes of H6′b protons could not be monitored due to overlap with other signals.

## Data Availability

The datasets used and analyzed during the current study are available from the corresponding authors upon request.

## Supplementary Materials

The supporting information can be downloaded at: https://www.mdpi.com/article/10.3390/ijms242015091/s1.

## References

[1] Brusilow S.W., Horwich A.L., Driver C.R., Beaded A.L., Sly W.S., Valle D. (2001). Urea cycle enzymes. The Metabolic and Molecular Bases of Inherited Disease.

[2] Brusilow S.W., Maestri N.E. (1996). Urea cycle disorders: Diagnosis, pathophysiology, and therapy. Adv. Pediatr..

[3] Walker V. (2014). Ammonia metabolism and hyperammonemic disorders. Adv. Clin. Chem..

[4] Häberle J., Boddaert N., Burlina A., Chakrapani A., Dixon M., Huemer M., Karall D., Martinelli D., Crespo P.S., Santer R. (2012). Suggested guidelines for the diagnosis and management of urea cycle disorders. Orphanet J. Rare Dis..

[5] Dover G.J., Brusilow S., Charache S. (1994). Induction of fetal hemoglobin production in subjects with sickle cell anemia by oral sodium phenylbutyrate. Blood.

[6] Basseri S., Lhotak S., Sharma A.M., Austin R.C. (2009). The chemical chaperone 4-phenylbutyrate inhibits adipogenesis by modulating the unfolded protein response. J. Lipid Res..

[7] Iannitti T., Palmieri B. (2011). Clinical and experimental applications of sodium phenylbutyrate. Drugs R D.

[8] Hayashi H., Mizuno T., Horikawa R., Nagasaka H., Yabuki T., Takikawa H., Sugiyama Y. (2012). 4-Phenylbutyrate modulates ubiquitination of hepatocanalicular MRP2 and reduces serum total bilirubin concentration. J. Hepatol..

[9] Upagupta C., Carlisle R.E., Dickhout J.G. (2017). Analysis of the potency of various low molecular weight chemical chaperones to prevent protein aggregation. Biochem. Biophys. Res. Commun..

[10] Enokida T., Yamasaki K., Okamoto Y., Taguchi K., Ishiguro T., Maruyama T., Seo H., Otagiri M. (2016). Tyrosine411 and arginine410 of human serum albumin play an important role in the binding of sodium 4-phenylbutyrate to site II. J. Pharm. Sci..

[11] European Medicines Agency Summary of Product Characteristics: Ammonaps. https://www.ema.europa.eu/en/documents/product-information/ammonaps-epar-product-information_en.pdf.

[12] Mennella J.A., Beauchamp G.K. (2008). Optimizing oral medications for children. Clin. Ther..

[13] Cramer F. (1954). Einschlussverbindungen.

[14] Frank S.G. (1975). Inclusion compounds. J. Pharm. Sci..

[15] Szejtli J. (1982). Cyclodextrins and Their Inclusion Complexes.

[16] Loftsson T., Brewster M.E. (2010). Pharmaceutical applications of cyclodextrins: Basic science and product development. J. Pharm. Pharmacol..

[17] Stella V.J., Rajewski R.A. (1997). Cyclodextrins: Their Future in Drug Formulation and Delivery. Pharm. Res..

[18] Loftsson T., Duchêne D. (2007). Cyclodextrins and their pharmaceutical applications. Int. J. Pharm..

[19] Commey K., Nakatake A., Enaka A., Nishi K., Tsukigawa K., Yamaguchi K., Ikeda H., Iohara D., Hirayama F., Otagiri M. (2023). Study of the inclusion complexes formed between 4-phenylbutyrate and α-, β- and γ-cyclodextrin in solution and evaluation on their taste-masking properties. J. Pharm. Pharmacol..

[20] Gadre A., Rüdiger V., Schneider H.-J., Connors K.A. (1997). Binding of cyclodextrins to alicyclic and aromatic substrates: Complex formation of α-, β-, and γ-cyclodextrins with substituted cyclohexanecarboxylic acids and phenylalkanoic acids. J. Pharm. Sci..

[21] Rekharsky M.V., Inoue Y. (1998). Complexation Thermodynamics of Cyclodextrins. Chem. Rev..

[22] Pauli W.A., Lach J.L. (1965). Interaction of pharmaceuticals with schardinger dextrins V. Interaction with a series of phenyl-substituted carboxylic acids. J. Pharm. Sci..

[23] Higuchi T., Connors K.A. (1965). Phase solubility techniques. Adv. Anal. Chem. Instrum..

[24] Rekharsky M.V., Mayhew M.P., Goldberg R.N., Ross P.D., Yamashoji Y., Inoue Y. (1997). Thermodynamic and Nuclear Magnetic Resonance Study of the Reactions of α- and β-Cyclodextrin with Acids, Aliphatic Amines, and Cyclic Alcohols. J. Phys. Chem. B.

[25] Uekama K., Hirayama F., Matsuo N., Koinuma H. (1978). Structural elucidation of the inclusion complexes of tolbutamide with α- and β-cyclodextrins in aqueous solution. Chem. Lett..

[26] Bender M.L., Komiyama M. (1978). Cyclodextrin Chemistry.

[27] Ikeda Y., Hirayama F., Arima H., Uekama K., Yoshitake Y., Harano K. (2004). NMR spectroscopic characterization of metoprolol/cyclodextrin complexes in aqueous solution: Cavity size dependency. J. Pharm. Sci..

[28] Schneider H.-J. (1991). Mechanisms of molecular recognition: Investigations of organic host-guest complexes. Angew. Chem. Int. Ed..

[29] Utsuki T., Hirayama F., Uekama K. (1993). Different photodimerization behavior of tranilast in α-, β- and γ-cyclodextrin complexes: Cavity-size and stoichiometry dependence. J. Chem. Soc. Perkin Trans. 2.

[30] Connors K.A. (1995). Population characteristics of cyclodextrin complex stabilities in aqueous solution. J. Pharm. Sci..

[31] Fernandes C.M., Carvalho R.A., da Costa S.P., Veiga F.J. (2003). Multimodal molecular encapsulation of nicardipine hydrochloride by β-cyclodextrin, hydroxypropyl-β-cyclodextrin and triacetyl-β-cyclodextrin in solution. Structural studies by 1H NMR and ROESY experiments. Eur. J. Pharm. Sci..

[32] Frisch M.J., Trucks G.W., Schlegel H.B., Scuseria G.E., Robb M.A., Cheeseman J.R., Scalmani G., Barone V., Petersson G.A., Nakatsuji H. (2016). “Gaussian 16”.

[33] Mineva T., Russo N., Sicilia E. (1998). Solvation effects on reaction profiles by the polarizable continuum model coupled with the Gaussian density functional method. J. Comp. Chem..

[34] Schneider H.-J., Hacket F., Rüdiger V., Ikeda H. (1998). NMR studies of cyclodextrins and cyclodextrin complexes. Chem. Rev..

[35] Redenti E., Szente L., Szejtli J. (2001). Cyclodextrin complexes of salts of acidic drugs. Thermodynamic properties, structural features, and pharmaceutical applications. J. Pharm. Sci..

[36] Job P. (1928). Formation and stability of inorganic complexes in solution. Ann. Chim..

[37] Al-Soufi W., Cabrer P.R., Jover A., Budal R.M., Tato J.V. (2003). Determination of second-order association constants by global analysis of 1H and 13C NMR chemical shifts: Application to the complexation of sodium fusidate and potassium helvolate by β- and γ-cyclodextrin. Steroids.

