# Supplementary material for: Study of the Structural Chemistry of the Inclusion Complexation of 4-Phenylbutyrate and Related Compounds with Cyclodextrins in Solution: Differences in Inclusion Mode with Cavity Size Dependency
Source: Int J Mol Sci. 2023 Oct 11;24(20):15091. doi: 10.3390/ijms242015091 (PMC10606765; doi:10.3390/ijms242015091)
Supplement: Supplementary file 1 [file ijms-24-15091-s001.zip › Supplementary file-proofread.pdf]

Study of the Structural Chemistry of the Inclusion Complexation of 4-Phenylbutyrate and Related Compounds with Cyclodextrins in Solution: Differences in Inclusion Mode with Cavity Size Dependency

Kindness L. Commey [1], Akari Nakatake [1], Airi Enaka [1], Ryota Nakamura [1], Koji Nishi [1,2], Kenji Tsukigawa [1,2], Hirohito Ikeda [3], Koki Yamaguchi [1,2], Daisuke Iohara [1,2], Fumitoshi Hirayama [1,2], Keishi Yamasaki [1,2,*] and Masaki Otagiri [1,2,*]

[1] Faculty of Pharmaceutical Sciences, Sojo University, 4-22-1 Ikeda, Kumamoto 860-0082, Japan; g1971d03@m.sojo-u.ac.jp (K.L.C.); chii6natoriumu2sou@gmail.com (A.N.); g1851033@m.sojo-u.ac.jp (A.E.); g1951079@m.sojo-u.ac.jp (R.N.); knishi@ph.sojo-u.ac.jp (K.N.); tsukigawa@ph.sojo-u.ac.jp (K.T.); kyamag05@ph.sojo-u.ac.jp (K.Y.); dio@ph.sojo-u.ac.jp (D.I.); fhira@ph.sojo-u.ac.jp (F.H.)
[2] DDS Research Institute, Sojo University, 4-22-1 Ikeda, Kumamoto 860-0082, Japan
[3] Faculty of Pharmaceutical Sciences, Fukuoka University, 8-19-1 Jonan-ku, Fukuoka 814-0180, Japan; ikeda@fukuoka-u.ac.jp (H.I.)
* Correspondence: kcyama@ph.sojo-u.ac.jp (K.Y.); otagirim@ph.sojo-u.ac.jp (M.O.)

Molecular modeling using the density functional theory (DFT)

DFT calculations were performed at B3PW91/cc-pVDZ level using the Gaussian 16 rev A.03 program (Gaussian Inc., USA) [32]. Using the possible poses created by MOE as the initial structure, they were structurally optimized in the gas phase. Each optimized structure calculated in the gas phase was further structurally optimized in water using the polarizable continuum model [33].

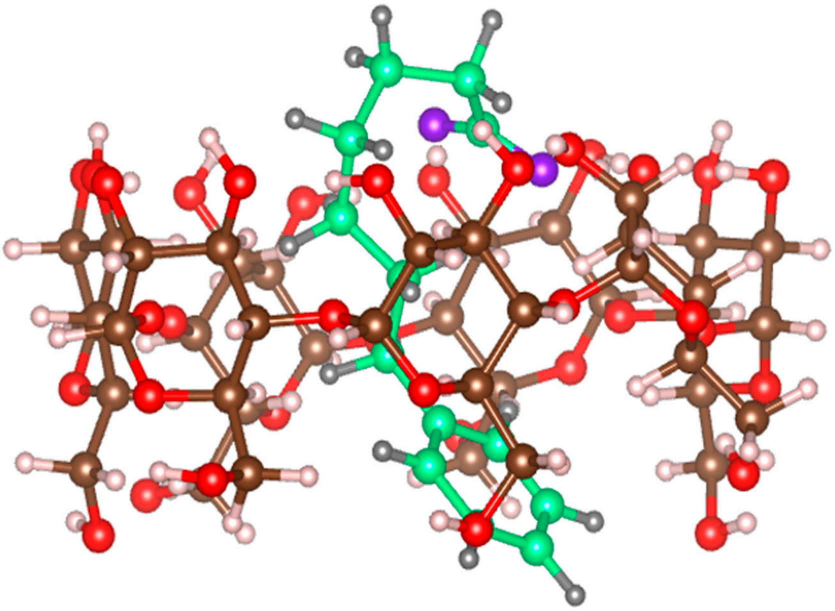

Figure S1. Possible inclusion structure of ionized PH (green ball and stick) and βCD estimated using the density functional theory. The upper and lower sides of the CD are the secondary and primary rims, respectively.

References

32. Frisch, M.J.; Trucks, G.W.; Schlegel, H.B.; Scuseria, G.E.; Robb, M.A.; Cheeseman, J.R.; Scalmani, G.; Barone, V.; Petersson, G.A.; Nakatsuji, H.; et al. *"Gaussian 16", Revision A.03*; Gaussian, Inc.: Wallingford, CT, USA, 2016.
33. Mineva, T.; Russo, N.; Sicilia, E. Solvation effects on reaction profiles by the polarizable continuum model coupled with the Gaussian density functional method. *J. Comp. Chem.* **1998**, *19*, 290–299. https://doi.org/10.1002/(SICI)1096-987X(199802)19:3<290::AID-JCC3>3.0.CO;2-O
